# Efficacy and safety of once-daily aclidinium in chronic obstructive pulmonary disease

**DOI:** 10.1186/1465-9921-12-55

**Published:** 2011-04-26

**Authors:** Paul W Jones, Stephen I Rennard, Alvar Agusti, Pascal Chanez, Helgo Magnussen, Leonardo Fabbri, James F Donohue, Eric D Bateman, Nicholas J Gross, Rosa Lamarca, Cynthia Caracta, Esther Garcia Gil

**Affiliations:** 1 St George's, University of London, London, UK; 2 Pulmonary and Critical Care Medicine, University of Nebraska Medical Center, Omaha, Nebraska, USA; 3 Thorax Institute, Hospital Clínic, Barcelona, and CIBER Enfermedades Respiratorias and Fundació Caubet-Cimera, Spain; 4 Département des Maladies Respiratoires, Université de la Mediterranée AP-HM, Marseille, France; 5 Pulmonary Research Institute at Hospital Grosshansdorf, Center for Pneumology and Thoracic Surgery, Grosshansdorf, Germany; 6 Department of Oncology, Haematology and Respiratory Diseases, University of Modena and Reggio Emilia, Modena, Italy; 7 Division of Pulmonary Disease and Critical Care Medicine, School of Medicine, University of North Carolina, Chapel Hill, North Carolina, USA; 8 Division of Pulmonology, Department of Medicine, University of Cape Town, Cape Town, South Africa; 9 Stritch-Loyola School of Medicine, Loyola University, Chicago, Illinois, USA; 10 Almirall, R&D Centre, Barcelona, Spain; 11 Forest Research Institute, New Jersey, USA

**Keywords:** Aclidinium bromide, anticholinergic, chronic obstructive pulmonary disease, long-acting muscarinic antagonist

## Abstract

**Background:**

The long-term efficacy and safety of aclidinium bromide, a novel, long-acting muscarinic antagonist, were investigated in patients with moderate to severe chronic obstructive pulmonary disease (COPD).

**Methods:**

In two double-blind, 52-week studies, ACCLAIM/COPD I (n = 843) and II (n = 804), patients were randomised to inhaled aclidinium 200 μg or placebo once-daily. Patients were required to have a post-bronchodilator forced expiratory volume in 1 second (FEV_1_)/forced vital capacity ratio of ≤70% and FEV_1 _<80% of the predicted value. The primary endpoint was trough FEV_1 _at 12 and 28 weeks. Secondary endpoints were health status measured by St George's Respiratory Questionnaire (SGRQ) and time to first moderate or severe COPD exacerbation.

**Results:**

At 12 and 28 weeks, aclidinium improved trough FEV_1 _versus placebo in ACCLAIM/COPD I (by 61 and 67 mL; both p < 0.001) and ACCLAIM/COPD II (by 63 and 59 mL; both p < 0.001). More patients had a SGRQ improvement ≥4 units at 52 weeks with aclidinium versus placebo in ACCLAIM/COPD I (48.1% versus 39.5%; p = 0.025) and ACCLAIM/COPD II (39.0% versus 32.8%; p = 0.074). The time to first exacerbation was significantly delayed by aclidinium in ACCLAIM/COPD II (hazard ratio [HR] 0.7; 95% confidence interval [CI] 0.55 to 0.92; p = 0.01), but not ACCLAIM/COPD I (HR 1.0; 95% CI 0.72 to 1.33; p = 0.9). Adverse events were minor in both studies.

**Conclusion:**

Aclidinium is effective and well tolerated in patients with moderate to severe COPD.

**Trial registration:**

ClinicalTrials.gov: NCT00363896 (ACCLAIM/COPD I) and NCT00358436 (ACCLAIM/COPD II).

## Background

Vagal cholinergic tone is the major reversible contributor to airway narrowing in chronic obstructive pulmonary disease (COPD); therefore, inhaled anticholinergic agents play a key role in the treatment of this disorder [1]. The Global Initiative for Chronic Obstructive Lung Disease (GOLD) recommends long-acting bronchodilators for the management of patients with stable COPD [2]; however, only one long-acting anticholinergic agent, tiotropium bromide, is available to date. Given the variability in individual patient responses to therapy in terms of efficacy and tolerability, and individual patient preferences for different formulations and delivery devices, the investigation of additional long-acting anticholinergic treatment options is warranted.

Aclidinium bromide is a novel, long-acting muscarinic antagonist currently in development for the treatment of COPD. Early studies in preclinical models indicated that aclidinium had potent bronchodilatory effects [3] and an inhibitory effect on mucus hypersecretion [4]. Preclinical and clinical studies have demonstrated the rapid hydrolysis of aclidinium in human plasma into inactive metabolites [5,6], suggesting a reduced propensity for systemic side effects. In Phase II studies, aclidinium showed long-lasting bronchodilatory activity and good tolerability with a low incidence of cardiovascular side effects [7,8].

This paper reports data from two Phase III studies, ACCLAIM/COPD (**AC**lidinium **CL**inical trial **A**ssessing efficacy and safety **I**n **M**oderate to severe **COPD **patients) I and II, to assess the long-term efficacy and safety of once-daily aclidinium 200 μg in patients with moderate to severe COPD.

## Methods

### Subjects

Male and non-pregnant, non-lactating female patients aged ≥40 years were included if they had a diagnosis of COPD according to GOLD criteria [2], with a post-bronchodilator forced expiratory volume in 1 second (FEV_1_)/forced vital capacity (FVC) ratio of ≤70% and FEV_1 _<80% of the predicted value [9]. The pre-dose FEV_1 _at randomisation had to be within 80-120% of the pre-bronchodilator FEV_1 _at screening. All patients were current or previous cigarette smokers with a smoking history of ≥10 pack-years. A previous history of exacerbations was not required.

Key exclusion criteria were: history or current diagnosis of asthma, allergic rhinitis or atopy; blood eosinophil count >600 cell/mm^3^; respiratory tract infection or COPD exacerbation within 6 weeks prior to screening or during the run-in period; hospitalisation for an acute COPD exacerbation within 3 months prior to screening; use of long-term oxygen therapy; clinically significant respiratory diseases other than COPD; unstable cardiac conditions.

Inhaled salbutamol was permitted on an as-needed basis, but had to be discontinued 6 hours prior to and during a study visit. The following concomitant COPD medications were allowed, provided their administration had been stable for at least 4 weeks prior to screening: inhaled corticosteroids or oral sustained-release theophyllines; oral or parenteral corticosteroids at maximal doses equivalent to 10 mg/day of prednisone or 20 mg every other day; oxygen therapy (<15 hours per day).

The studies were performed in accordance with the Declaration of Helsinki, International Conference on Harmonisation Good Clinical Practice Guidelines and local regulations. Prior to study initiation at each centre, the protocol was approved by an Independent Ethics Committee or Institutional Review Board. All patients gave written informed consent. The studies were registered with ClinicalTrials.gov as follows: NCT00363896 (ACCLAIM/COPD I) and NCT00358436 (ACCLAIM/COPD II).

### Study design

Two identical 52-week, double-blind, randomised, placebo-controlled, parallel-group studies were performed as regulatory organisations require replicated data. ACCLAIM/COPD I was conducted at 139 centres in 16 European countries and ACCLAIM/COPD II at 119 sites in 7 countries (primarily in North America). After screening, patients underwent a 14-day run-in period to assess their disease stability. Eligible patients were randomised in a 3:1 ratio to receive aclidinium 200 μg or matching placebo once-daily via the Genuair^® ^inhaler, a novel multidose dry powder inhaler [10].

It was estimated that a total sample size of 820 patients per study (615 and 205 patients in the aclidinium and placebo arms, respectively) would provide at least 90% power to detect a difference of 100 mL in trough FEV_1 _between the two arms at 12 and 28 weeks, with a two-sided 5% level of significance and assuming a standard deviation of 310 mL [7].

### Assessments

Spirometry was conducted according to American Thoracic Society (ATS)/European Respiratory Society (ERS) recommendations [11] at 1 hour pre-dose and immediately before dosing during study visits on Day 1 (baseline); Day 2; Week 1; every month up to Week 20; and thereafter every 2 months until Week 52. Measurements were also performed at 0.25, 0.5, 1, 2 and 3 hours post-dose on Day 1; and at 0.5, 1, 2 and 3 hours post-dose at Weeks 1, 4, 8, 12, 28, 44 and 52. Sites were provided with identical spirometry equipment, a detailed study manual and training. A centralised quality-assurance review of all spirometry data was conducted throughout the study. The spirometry data were electronically transmitted to a data-management centre where an independent, blinded, spirometric expert reviewed the acceptability and repeatability of the data according to ATS/ERS acceptability criteria.

Health status and dyspnoea were evaluated pre-dose on Day 1 (baseline) and at Weeks 12, 28, 44 and 52 using the St George's Respiratory Questionnaire (SGRQ; self administered) and Baseline/Transitional Dyspnoea Index (BDI/TDI; administered by an independent reviewer).

Data on morning and evening peak expiratory flow, COPD symptoms over 24 hours (breathlessness on a scale of 0 to 4; cough, sputum production and wheezing on a scale of 0 to 3), use of daily rescue medication and concomitant medication were recorded in the patient's diary and reviewed by the investigator at each visit to identify the occurrence of a COPD exacerbation. COPD exacerbations were defined as an increase in COPD symptoms over at least two consecutive days, associated with increased use of bronchodilators (mild exacerbation), treatment with antibiotics and/or systemic corticosteroids (moderate exacerbation) or leading to hospitalisation (severe exacerbation).

Safety was assessed by adverse-event (AE) monitoring, physical examination, blood pressure, 12-lead electrocardiogram (ECG) and laboratory data (haematology, biochemistry and urinalysis).

### Statistical analysis

Efficacy analyses were performed on the intent-to-treat population, defined as all randomised patients who received at least one dose of study medication and who had a baseline and at least one post-baseline trough FEV_1 _measurement. The safety population consisted of all randomised patients who received at least one dose of study medication.

FEV_1_, FVC, inspiratory capacity, peak expiratory flow and change from baseline in SGRQ scores were analysed using an analysis of covariance (ANCOVA) model with treatment and sex as factors, and baseline values and age as covariates; the same model, without adjustment for age and sex, was used to analyse the change from baseline in dyspnoea, COPD symptom scores and use of daily rescue medication. Missing data were imputed using a last observation carried forward approach. The minimal clinically important difference (MCID) for the SGRQ and TDI is 4 units and 1 unit, respectively [12,13]. A logistic regression model was used to analyse the number of patients with a ≥4-unit decrease in SGRQ total score (with adjustment for age, sex and baseline values) and a ≥1-unit improvement in TDI total score (with adjustment for baseline values). Time to first moderate or severe COPD exacerbation was evaluated using a Cox Proportional Hazards model adjusted for age, sex and COPD severity at screening.

The primary endpoint was trough FEV_1 _at Weeks 12 and 28 to fulfil US and European regulatory requirements, respectively. Secondary endpoints were: the number of patients who achieved a clinically relevant improvement in health-related quality of life at 52 weeks, as measured by a ≥4-unit decrease from baseline on the SGRQ total score; and time to first moderate or severe COPD exacerbation.

## Results

### Study population

**Figure 1:**
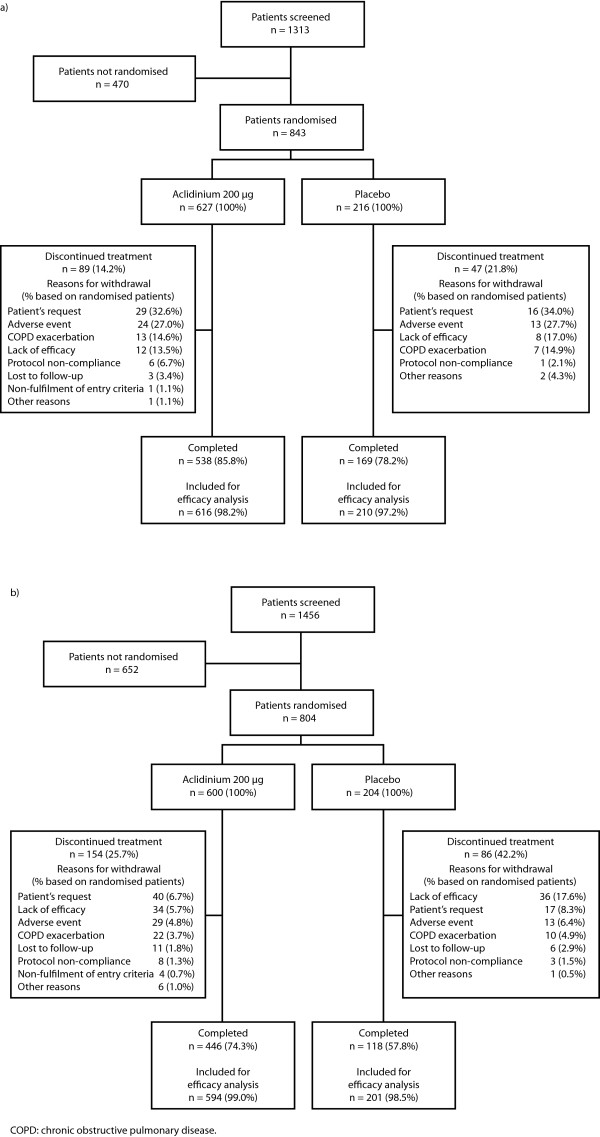
Patient disposition in a) ACCLAIM/COPD I, and b) ACCLAIM/COPD II

Patient disposition in the two studies is illustrated in Figure 1. Table 1 shows demographics and disease status at baseline. Compared with ACCLAIM/COPD I, in ACCLAIM/COPD II, there were slightly more females and fewer smokers, the post-bronchodilator FEV_1 _was slightly lower and the bronchodilator reversibility was slightly higher. The percentage of patients who continued to use inhaled corticosteroids in the aclidinium and placebo groups was 39.1% and 40.0%, respectively, in ACCLAIM/COPD I, and 40.2% and 44.3%, respectively, in ACCLAIM/COPD II. In both studies, a lower proportion of patients discontinued treatment in the aclidinium arm compared with the placebo arm; the difference in withdrawal rates between the study arms was greater in ACCLAIM/COPD II (25.7% versus 42.2%, respectively; 16.5% difference) than in ACCLAIM/COPD I (14.2% versus 21.8%, respectively; 7.6% difference).

**Table 1:** Baseline demographics and disease status (safety population)

	ACCLAIM/COPD I		ACCLAIM/COPD II	
	
	Aclidinium 200 μg (n = 627)	Placebo (n = 216)	Aclidinium 200 μg (n = 600)	Placebo (n = 204)
Age, mean (SD) years	62.6 (8.2)	61.9 (8.3)	65.1 (8.6)	65.2 (8.6)
Male, n (%)	488 (77.8)	175 (81.0)	383 (63.8)	124 (60.8)
Caucasian, n (%)	627 (100.0)	215 (99.5)	552 (92.0)	189 (92.7)
BMI, mean (SD), kg/m^2^	26.4 (4.70)	26.7 (4.65)	26.8 (5.9)	26.6 (6.1)
Current smoker, n (%)	283 (45.1)	98 (45.4)	222 (37.0)	79 (38.7)
Smoking history, mean (SD) pack-years	40.4 (21.0)	38.4 (18.3)	57.8 (29.9)	58.2 (28.4)
Post-bronchodilator FEV_1_, mean (SD) % of predicted value	54.2 (15.1)	52.9 (15.2)	50.6 (15.6)	49.4 (15.1)
Post-bronchodilator FEV_1_/FVC ratio, mean (SD) %	49.2 (11.4)	48.9 (11.1)	48.0 (11.7)	47.1 (11.9)
Bronchodilator reversibility, mean (SD) %	12.3 (14.2)	12.9 (14.0)	17.7 (15.7)	17.3 (13.5)
Pre-dose FEV_1 _on Day 1, mean (SD) L	1.413 (0.514)	1.385 (0.509)	1.214 (0.489)	1.154 (0.479)
SGRQ total score, mean (SD)	47.3 (17.6)	47.3 (18.0)	45.2 (16.8)	47.1 (16.3)
Baseline Dyspnoea Index focal score, mean (SD)	6.3 (2.2)	6.4 (2.2)	6.5 (2.3)	6.2 (2.3)
Pre-study COPD medication, n (%)	532 (84.9)	178 (82.4)	505 (84.2)	177 (86.8)
Inhaled corticosteroids	239 (38.1)	83 (38.4)	228 (38.0)	78 (38.2)
Long-acting β_2_-agonists	99 (15.8)	34 (15.7)	38 (6.3)	14 (16.9)
Long-acting β_2_-agonists + inhaled corticosteroids*	170 (27.1)	47 (21.8)	217 (36.2)	76 (27.3)
Long-acting muscarinic antagonist	110 (17.5)	39 (18.1)	103 (17.2)	37 (18.1)
Short-acting β_2_-agonists	331 (52.8)	95 (44.0)	408 (68.0)	143 (70.1)
Short-acting β_2_-agonists + short-acting muscarinic agents	98 (15.6)	45 (20.8)	55 (9.2)	25 (12.3)
Short-acting muscarinic agents	99 (15.8)	26 (12.0)	51 (8.5)	28 (13.7)
Xanthines	116 (18.5)	34 (15.7)	29 (4.8)	6 (2.9)
Patients with ≥1 self-reported COPD exacerbation in previous year, n (%)^†^	354 (56.5)	131 (60.6)	182 (30.3)	64 (31.4)

*The percentages of patients who received long-acting β_2_-agonists plus inhaled corticosteroids include those who switched from long-acting β_2_-agonists plus inhaled corticosteroids to inhaled corticosteroids prior to screening.

^†^Data missing for four patients in ACCLAIM/COPD I (two each in the aclidinium and placebo arms).

BMI: body mass index; COPD: chronic obstructive pulmonary disease; FEV_1_: forced expiratory volume in 1 second; FVC: forced vital capacity; SGRQ: St George's Respiratory Index; SD: standard deviation.

### Efficacy

#### Lung function

**Figure 2:**
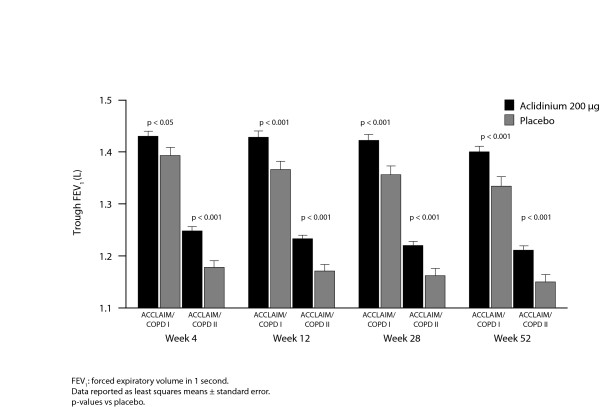
Trough forced expiratory volume in 1 second for aclidinium 200 μg versus placebo over 52 weeks in ACCLAIM/COPD I and II

At Weeks 12 and 28, aclidinium improved the baseline-adjusted mean trough FEV_1 _compared with placebo in ACCLAIM/COPD I (by 61 and 67 mL, respectively; both p < 0.001) and ACCLAIM/COPD II (by 63 and 59 mL, respectively; both p < 0.001). In both studies, this effect was maintained over the 52-week study period (Figure 2; Table 2). In ACCLAIM/COPD I, treatment differences in trough FEV_1 _ranged from 60 to 67 mL at all time points except for Weeks 1 and 4 where the differences were 44 and 37 mL, respectively (p-values ranged from 0.021 to <0.0001). In ACCLAIM/COPD II, treatment differences ranged from 51 to 78 mL throughout the study (p-values ranged from 0.0011 to <0.0001).

**Table 2:** Baseline-adjusted mean differences between aclidinium and placebo on lung function parameters at all measured time points throughout the 52-week studies

Parameter	ACCLAIM/COPD I	ACCLAIM/COPD II
Trough FEV_1_, mL	37-67*	51-78^§^
Peak FEV_1_, mL	147-177^‡^	141-156^‡^
AUC_(0-3 h)_, mL	135-166^‡^	133-150^‡^
Trough FVC, mL	96-141^§^	83-148^†^
Peak FVC, mL	275-310^‡^	259-315^‡^
Trough IC, mL	58-101*	64-135^§^
Peak IC, mL	166-204^‡^	171-220^‡^
Morning PEF, mL/min	6.1-13.9*	9.8-17.2*
Evening PEF, mL/min	18.9-22.8^‡^	16.9-22.9^§^

*p < 0.05; ^§^p < 0.01; ^†^p < 0.001; ^‡^p < 0.0001.

Data are reported as the minimum and maximum values over the treatment period; the indicated p-values apply throughout the stated range.

FEV_1_: forced expiratory volume in 1 second; AUC_(0-3 h)_: area under the curve from 0 to 3 h post-dose; FVC: forced vital capacity; IC: inspiratory capacity; PEF: peak expiratory flow.

Significant improvements in other lung function parameters were observed for aclidinium versus placebo throughout the two studies (Table 2). In both studies, the median time to peak FEV_1 _in the aclidinium group was 2 hours and significantly more patients had an increase in FEV_1 _of ≥15% above baseline at 0.25, 0.5, 1, 2 and 3 hours post-dose with aclidinium compared with placebo (p < 0.0001 at each time point).

An exploratory post-hoc analysis using pooled data from the two studies was conducted to examine the treatment differences in trough FEV_1 _when the patient population was stratified according to the use of concomitant inhaled corticosteroids. The magnitude of improvement in trough FEV_1 _with aclidinium over placebo was slightly greater in patients who used inhaled corticosteroids compared with those who did not at Weeks 4 (64 versus 47 mL), 44 (80 versus 48 mL) and 52 (90 versus 48 mL). However, treatment differences in trough FEV_1 _were slightly lower or similar in patients who used inhaled corticosteroids compared with those who did not at Weeks 12 (52 versus 68 mL) and 28 (65 versus 63 mL).

#### Quality of life and dyspnoea

**Figure 3:**
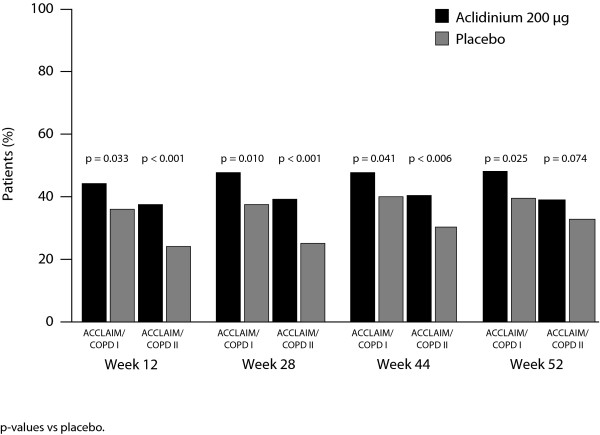
Percentage of patients with a clinically relevant improvement in St George's Respiratory Questionnaire total score (≥4 units) over time in ACCLAIM/COPD I and II

In ACCLAIM/COPD I, significantly more patients receiving aclidinium had an improvement in SGRQ total score ≥4 units compared with placebo at all measured time points; at 52 weeks, the percentage of patients achieving this improvement was 48.1% versus 39.5% for aclidinium versus placebo, respectively (p = 0.025; Figure 3). In ACCLAIM/COPD II, significantly more patients had an improvement in SGRQ total score ≥4 units with aclidinium versus placebo up to 44 weeks; at 52 weeks, the percentage of patients achieving this improvement was 39.0% versus 32.8% for aclidinium versus placebo, respectively (p = 0.074; Figure 3). At Week 52, the mean improvement from baseline in SGRQ total score was greater for aclidinium compared with placebo in ACCLAIM/COPD I (-4.63 versus -3.10, respectively; treatment difference 1.53; p = 0.19) and ACCLAIM/COPD II (-3.49 versus -1.28, respectively; treatment difference 2.21; p = 0.021). Greater improvements in baseline-adjusted SGRQ total score were observed for aclidinium versus placebo throughout both ACCLAIM/COPD I (range 1.53-2.71; p < 0.05 at Weeks 12 and 28) and ACCLAIM/COPD II (range 2.21-3.54; p < 0.05 at all time points).

In ACCLAIM/COPD I, more patients receiving aclidinium versus placebo exceeded the MCID for TDI focal score at 52 weeks (56.4% versus 38.0%; odds ratio 2.22; 95% confidence interval [CI] 1.57 to 3.13; p < 0.0001); similar results were observed at Weeks 12, 28 and 44. The proportion of patients who exceeded the MCID was also greater for aclidinium versus placebo at all time points in ACCLAIM/COPD II, but was significant only at Week 12 (53.1% versus 42.7%; odds ratio 1.53; 95% CI 1.04 to 2.23; p = 0.029). At Week 52, the mean improvement from baseline in TDI focal score was greater for aclidinium compared with placebo in ACCLAIM/COPD I (1.83 versus 0.61; treatment difference 1.22; p < 0.0001) and ACCLAIM/COPD II (1.61 versus 1.09; treatment difference 0.52; p = 0.12). Baseline-adjusted mean treatment differences in TDI focal score favoured aclidinium throughout the study in ACCLAIM/COPD I (range 0.73-1.22; p < 0.01 at all time points) and ACCLAIM/COPD II (range 0.52-1.03; p < 0.01 at Weeks 12, 28 and 44).

#### Exacerbations

**Figure 4:**
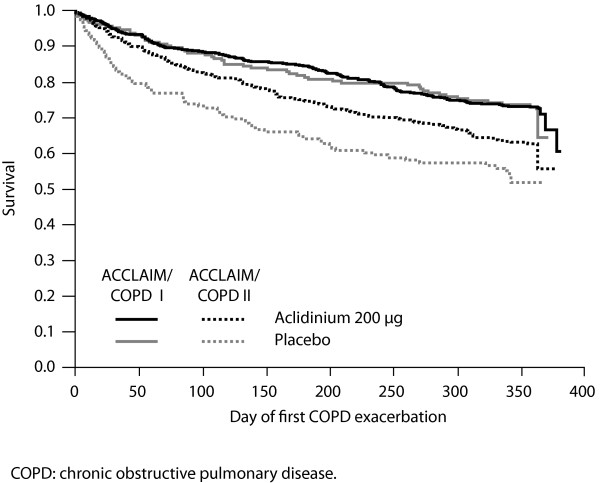
Kaplan-Meier survival curves for the time to first moderate or severe chronic obstructive pulmonary disease exacerbation in ACCLAIM/COPD I and II

The rate of moderate or severe exacerbations in the placebo arm was numerically lower in ACCLAIM/COPD I (0.46 events/patient/year, respectively) than in ACCLAIM/COPD II (0.80 events/patient/year, respectively). In ACCLAIM/COPD I, the proportion of patients who experienced a moderate or severe exacerbation was similar in the aclidinium and placebo groups (26.6% versus 25.7%, respectively). There was no significant difference between the two groups in time to first moderate or severe exacerbation (hazard ratio 1.0; 95% CI 0.72 to 1.33; p = 0.9; Figure 4). In ACCLAIM/COPD II, fewer patients in the aclidinium group experienced a moderate or severe exacerbation compared with those in the placebo group (33.2% versus 39.8%, respectively; rate ratio 0.7; 95% CI 0.55 to 0.90; p = 0.0046). Aclidinium significantly delayed the time to first moderate or severe exacerbation compared with placebo (hazard ratio 0.7; 95% CI 0.55 to 0.92; p = 0.01; Figure 4).

#### Other efficacy measures

In ACCLAIM/COPD I, there were no significant differences between aclidinium and placebo in patient-recorded daily symptom scores for breathlessness, cough, sputum production or wheezing, or use of daily rescue medication. However, in ACCLAIM/COPD II, there were significant differences in favour of aclidinium for cough and sputum production (Week 1), breathlessness (Week 12), wheezing (Week 28) and use of daily rescue medication (Weeks 12 and 28) (p < 0.05 for all).

### Safety

The overall incidence of AEs was similar in the aclidinium and placebo groups for ACCLAIM/COPD I and II (Table 3). AEs, most of which were mild to moderate in intensity, were similar between the two groups in both studies. AEs associated with anticholinergic medication occurred with an incidence of <1% in both the aclidinium and placebo groups, except for dry mouth (1.0% versus 0.9% respectively) in ACCLAIM/COPD I, and urinary tract infection (4.8% versus 4.9%, respectively), constipation (2.2% versus 2.0%, respectively) and dry mouth (0.3% versus 1.5%, respectively) in ACCLAIM/COPD II. AEs reported by ≥2% of patients in either group of either study and possible anticholinergic AEs are provided in Tables 4 and 5, respectively.

**Table 3:** Adverse-event profile in ACCLAIM/COPD I and II

	ACCLAIM/COPD I		ACCLAIM/COPD II	
	Aclidinium 200 μg (n = 627) n (%)	Placebo (n = 216) n (%)	Aclidinium 200 μg (n = 600) n (%)	Placebo (n = 204) n (%)
Total AEs	355 (56.6)	128 (59.3)	479 (79.8)	154 (75.5)
SAEs	50 (8.0)	22 (10.2)	62 (10.3)	23 (11.3)
Deaths	7 (1.1)	4 (1.9)	6 (1.0)	3 (1.5)
Cardiac disorders*	32 (5.1)	14 (6.5)	41 (6.8)	17 (8.3)
Vascular disorders*	22 (3.5)	14 (6.5)	34 (5.7)	12 (5.9)

*System organ classes.

COPD exacerbations were reported as part of the efficacy evaluations and, unless life-threatening, were not considered to be AEs.

AEs: adverse events; COPD: chronic obstructive pulmonary disorder; SAEs: serious adverse events.

**Table 4:** Adverse events reported by ≥2% of patients in any treatment group in ACCLAIM/COPD I or ACCLAIM/COPD II

Adverse event preferred term	ACCLAIM/COPD I		ACCLAIM/COPD II	
	
	Aclidinium 200 μg (n = 627) n (%)	Placebo (n = 216) n (%)	Aclidinium 200 μg (n = 600) n (%)	Placebo (n = 204) n (%)
Nasopharyngitis	102 (16.3)	31 (14.4)	76 (12.7)	23 (11.3)
Headache	71 (11.3)	27 (12.5)	85 (14.2)	26 (12.7)
Upper respiratory tract infection	8 (1.3)	3 (1.4)	65 (10.8)	20 (9.8)
Back pain	30 (4.8)	10 (4.6)	40 (6.7)	15 (7.4)
Diarrhoea	17 (2.7)	1 (0.5)	42 (7.0)	8 (3.9)
Pharyngolaryngeal pain	21 (3.3)	3 (1.4)	25 (4.2)	11 (5.4)
Urinary tract infection	5 (0.8)	2 (0.9)	29 (4.8)	10 (4.9)
Cough	29 (4.6)	8 (3.7)	29 (4.8)	8 (3.9)
Hypertension	9 (1.4)	9 (4.2)	27 (4.5)	6 (2.9)
Sinusitis	5 (0.8)	1 (0.5)	27 (4.5)	9 (4.4)
Dyspnoea	12 (1.9)	5 (2.3)	17 (2.8)	9 (4.4)
Arthralgia	17 (2.7)	1 (0.5)	24 (4.0)	5 (2.5)
Influenza	21 (3.3)	4 (1.9)	22 (3.7)	8 (3.9)
Pain in extremity	5 (0.8)	3 (1.4)	23 (3.8)	4 (2.0)
Dizziness	15 (2.4)	6 (2.8)	19 (3.2)	7 (3.4)
Abdominal pain	6 (1.0)	3 (1.4)	20 (3.3)	0 (0.0)
Muscle spasms	4 (0.6)	0 (0.0)	19 (3.2)	5 (2.5)
Musculoskeletal pain	7 (1.1)	1 (0.5)	19 (3.2)	3 (1.5)
Pneumonia	12 (1.9)	7 (3.2)	10 (1.7)	4 (2.0)
Rhinitis	12 (1.9)	7 (3.2)	14 (2.3)	4 (2.0)
Myalgia	3 (0.5)	3 (1.4)	18 (3.0)	3 (1.5)
Oedema peripheral	7 (1.1)	2 (0.9)	18 (3.0)	2 (1.0)
Chest pain	5 (0.8)	1 (0.5)	10 (1.7)	6 (2.9)
Insomnia	7 (1.1)	1 (0.5)	8 (1.3)	6 (2.9)
Nausea	8 (1.3)	3 (1.4)	16 (2.7)	6 (2.9)
Viral infection	9 (1.4)	6 (2.8)	5 (0.8)	3 (1.5)
Bronchitis	4 (0.6)	1 (0.5)	10 (1.7)	5 (2.5)
Depression	3 (0.5)	3 (1.4)	15 (2.5)	4 (2.0)
Blood glucose increased	3 (0.5)	4 (1.9)	3 (0.5)	5 (2.5)
Epistaxis	5 (0.8)	0 (0.0)	8 (1.3)	5 (2.5)
Neck pain	7 (1.1)	0 (0.0)	6 (1.0)	5 (2.5)
Procedural pain	1 (0.2)	1 (0.5)	5 (0.8)	5 (2.5)
Vomiting	7 (1.1)	0 (0.0)	15 (2.5)	5 (2.5)
Contusion	11 (1.8)	3 (1.4)	14 (2.3)	1 (0.5)
Rash	2 (0.3)	0 (0.0)	14 (2.3)	3 (1.5)
Toothache	12 (1.9)	5 (2.3)	12 (2.0)	2 (1.0)
Constipation	4 (0.6)	0 (0.0)	13 (2.2)	4 (2.0)
Gamma-glutamyl transferase increased	13 (2.1)	2 (0.9)	2 (0.3)	3 (1.5)
Angina pectoris	4 (0.6)	3 (1.4)	3 (0.5)	4 (2.0)
Fatigue	4 (0.6)	1 (0.5)	10 (1.7)	4 (2.0)
Lower respiratory tract infection	5 (0.8)	0 (0.0)	5 (0.8)	4 (2.0)
Musculoskeletal chest pain	7 (1.1)	1 (0.5)	12 (2.0)	4 (2.0)
Pain	0 (0.0)	0 (0.0)	6 (1.0)	4 (2.0)
Pyrexia	8 (1.3)	3 (1.4)	12 (2.0)	1 (0.5)
Rhinorrhoea	2 (0.3)	1 (0.5)	9 (1.5)	4 (2.0)
Syncope	1 (0.2)	0 (0.0)	2 (0.3)	4 (2.0)

**Table 5:** Patients with ≥2 possible anticholinergic adverse events in any group in ACCLAIM/COPD I or ACCLAIM/COPD II (by system organ class and preferred term)

System organ class	Adverse event preferred term	ACCLAIM/COPD I		ACCLAIM/COPD II	
		
		Aclidinium 200 μg (n = 627) n (%)	Placebo (n = 216) n (%)	Aclidinium 200 μg (n = 600) n (%)	Placebo (n = 204) n (%)
	Atrial fibrillation	3 (0.5)	2 (0.9)	3 (0.5)	0 (0.0)
	Atrial flutter	0 (0.0)	0 (0.0)	2 (0.3)	1 (0.5)
Cardiac disorders	Sinus tachycardia	2 (0.3)	1 (0.5)	1 (0.2)	0 (0.0)
	Tachycardia	3 (0.5)	0 (0.0)	3 (0.5)	1 (0.5)
	Ventricular extrasystoles	1 (0.2)	1 (0.5)	3 (0.5)	0 (0.0)

	Dry eye	1 (0.2)	1 (0.5)	5 (0.8)	0 (0.0)
	Eye irritation	0 (0.0)	0 (0.0)	3 (0.5)	1 (0.5)
Eye disorders	Eye pain	3 (0.5)	0 (0.0)	0 (0.0)	0 (0.0)
	Vision blurred	0 (0.0)	0 (0.0)	3 (0.5)	1 (0.5)
	Visual acuity reduced	0 (0.0)	0 (0.0)	2 (0.3)	1 (0.5)

Gastrointestinal disorders	Constipation	4 (0.6)	0 (0.0)	13 (2.2)	4 (2.0)
	Dry mouth	6 (1.0)	2 (0.9)	2 (0.3)	3 (1.5)

Infections and infestations	Urinary tract infection	0 (0.0)	0 (0.0)	29 (4.8)	10 (4.9)

The incidence of serious adverse events (SAEs) was similar for aclidinium and placebo in both studies (Table 3). In ACCLAIM/COPD I, only two patients, both in the aclidinium group, reported SAEs that were considered to be treatment-related (angle closure glaucoma and open angle glaucoma). Six patients in ACCLAIM/COPD II reported treatment-related SAEs, of whom four received aclidinium (atrial fibrillation and sick sinus syndrome, atrial flutter, myocardial infarction and pneumonia) and two received placebo (headache and rash [same patient], and cerebellar infarction). In each study, the percent incidence of deaths was lower in the aclidinium group than in the placebo group (Table 3), and none were considered to be treatment-related.

Cardiac and vascular disorders were reported at a similar frequency in the aclidinium and placebo groups of each study (Table 3). In ACCLAIM/COPD I, only two patients (one in each group) experienced a cerebrovascular accident or ischaemic stroke. Nine patients in ACCLAIM/COPD II reported a cerebrovascular accident, cerebral infarction or transient ischaemic attack, of whom seven (1.2%) received aclidinium and two (1.0%) received placebo.

No clinically relevant changes in laboratory parameters, vital signs or ECG results (including QTc intervals) were observed in either group in either study.

## Discussion

These studies show that aclidinium 200 μg once-daily improves lung function in patients with moderate to severe COPD. Compared with placebo, aclidinium improved the adjusted mean trough FEV_1 _by 60 to 67 mL at all time points after 4 weeks in ACCLAIM/COPD I, and by 51 to 78 mL throughout the treatment period in ACCLAIM/COPD II. The slight decrease in the trough FEV_1 _response with aclidinium over time does not appear to be related to tachyphylaxis, as a similar decline occurred with placebo in both studies. There were some small differences in the patient demographics between the studies, perhaps the most relevant being a lower mean bronchodilator reversibility in ACCLAIM/COPD I compared with ACCLAIM/COPD II. Despite this difference, the improvements in trough FEV_1 _were similar. These trough FEV_1 _improvements are smaller than those seen in previous studies of aclidinium 200 μg [7] and tiotropium [14-20], where the magnitude of improvement ranged from 100 to 150 mL. Interestingly, in the recent UPLIFT trial (n = 5993), reported differences in trough FEV_1 _between tiotropium and placebo were smaller, ranging from 87 to 103 mL [21]. In the ACCLAIM/COPD and UPLIFT trials, patients were allowed to use inhaled corticosteroids as concomitant therapy, while in UPLIFT, they could also use long-acting β_2_-agonists. The UPLIFT authors speculated that concomitant therapy may have diminished the measured treatment effect [21], and it is possible that a similar effect operated in the ACCLAIM/COPD trials, although perhaps to a lesser extent, given that long-acting β-agonists were not permitted in these studies. This hypothesis cannot be tested reliably by a subgroup analysis to compare patients with or without concomitant inhaled corticosteroids, because it is not possible to remove the potential confounding effect of underlying disease severity having influenced the physician's decision to treat with inhaled corticosteroids. Pooled post-hoc analyses of the ACCLAIM/COPD studies suggested that patients who used inhaled corticosteroids during aclidinium treatment showed greater bronchodilation than those who did not, but these differences were not consistent over time.

As FEV_1 _correlates poorly with health status and COPD symptoms [22], it is important to directly investigate the effects of therapy on these parameters. At all time points in ACCLAIM/COPD I, significantly greater proportions of patients with aclidinium versus placebo exceeded the MCID for SGRQ total score and TDI focal score, and therefore may be considered to have achieved clinically significant improvements in health status and dyspnoea. In ACCLAIM/COPD II, significantly greater proportions of patients with aclidinium versus placebo exceeded the MCID for SGRQ total score up to Week 44 and TDI focal score at Week 12. The lack of a statistically significant difference in SGRQ total score at Week 52 in ACCLAIM/COPD II may be due to the higher rate of dropouts with placebo versus aclidinium, resulting in a greater proportion of 'healthy survivors' remaining in the placebo arm and reducing the treatment differences observed with aclidinium. In Figure 3, it can be seen that the percentage of placebo-treated patients with an SGRQ improvement ≥4 units increased over time in ACCLAIM/COPD II (24.1-32.8%), but remained relatively constant in ACCLAIM/COPD I (36.0-40.0%).

Aclidinium significantly delayed the time to first moderate or severe exacerbation compared with placebo in ACCLAIM/COPD II, but not in ACCLAIM/COPD I. A possible reason for this variation may be the lower rate of moderate or severe exacerbations in the placebo group in ACCLAIM/COPD I compared with ACCLAIM/COPD II (0.46 versus 0.80 events/patient/year, respectively). The placebo exacerbation rate in ACCLAIM/COPD II was similar to that observed in a 1-year study of tiotropium (0.95 events/patient/year) [15] and in the UPLIFT study (0.85 events/patient/year) [21], both of which also reported significant treatment effects on exacerbations. As significant effects on exacerbation are more likely to be shown in populations with frequent exacerbations, the low exacerbation rate in ACCLAIM/COPD I may explain the lack of difference observed between aclidinium and placebo. It should be noted that the study was not powered for exacerbations and the population was not enriched by recruiting patients with a history of exacerbation in the year before entry. The difference in rates also cannot be explained entirely in terms of demographic differences, chiefly a higher proportion of women and a greater smoking history in ACCLAIM/COPD II. While the reasons remain unknown, we postulate that the difference may be related to the fact that exacerbations were defined based on healthcare utilisation (i.e. moderate exacerbation; use of oral corticosteroids and/or antibiotics; severe exacerbation; requirement for in-patient hospital treatment); however, differences in healthcare resource utilisation do not appear to have caused any disparity in exacerbation rates in previous studies conducted in different territories.

In accordance with previous Phase I/II studies [6-8,23], aclidinium was safe and well tolerated in these Phase III studies. The percentages of SAEs, cardiovascular events and deaths with aclidinium were either similar or lower than those with placebo. Pooled analyses of safety data from tiotropium trials have indicated that, compared with placebo, tiotropium is associated with an excess risk of dry mouth, urinary retention and arrhythmias [24,25]. In the ACCLAIM/COPD studies, the incidence of anticholinergic AEs was similar between the two groups. Although safety analyses of aclidinium have not yet been conducted across multiple studies in a pooled analysis as for tiotropium, the initial evidence suggests that aclidinium may offer a low potential for class-related systemic side effects.

## Conclusions

Treatment with aclidinium 200 μg once-daily is safe and improves lung function and symptomatic endpoints in patients with moderate or severe COPD. The clinical relevance of the observed improvements in trough FEV_1 _is uncertain, as a minimum clinically important difference in trough FEV_1 _has not yet been established. Some authors have suggested an improvement of about 100-120 mL as a possible benchmark, but this is expert opinion rather than a validated parameter [22,26]. There is evidence that treatment differences in trough FEV_1 _values have been decreasing in recent trials due to changes in the availability of concomitant medications and baseline patient populations over time [27]. Regulators may consider a change of 5-10% from baseline FEV_1 _to be clinically meaningful [22]; assuming a baseline FEV_1 _of 1.29 L (the mean across treatment arms in this study), this corresponds to an increase of 64-129 mL. Additional clinical studies of aclidinium are ongoing in patients with moderate to severe COPD to investigate both higher and/or twice-daily dosing that could offer improvements on the efficacy profile compared with the 200 μg once-daily dose. In a Phase II, placebo- and active comparator-controlled study, aclidinium 400 μg twice-daily provided bronchodilation over 24 hours that was statistically superior and clinically meaningful compared with placebo, and comparable with tiotropium 18 μg once-daily [28]. Early Phase III data suggest that aclidinium 200 μg and 400 μg twice-daily for 12 weeks both provide statistically and clinically significant improvements in lung function compared with placebo [29].

## List of abbreviations used

AE: adverse event; ANCOVA: analysis of covariance; ATS: American Thoracic Society; BDI: Baseline Dyspnoea Index; CI: confidence interval; COPD: chronic obstructive pulmonary disease; ECG: electrocardiogram; ERS: European Respiratory Society; FEV_1_: forced expiratory volume in 1 second; FVC: forced vital capacity; HR: hazard ratio; MCID: minimal clinically important difference; SAE: serious adverse event; SGRQ: St George's Respiratory Questionnaire; TDI: Transitional Dyspnoea Index

## Competing interests

PWJ has received fees from a number of pharmaceutical companies, including Almirall, for speaking at meetings and for consulting and participating in advisory board meetings, and has also received support for research from GSK.

SIR has consulted on or participated in advisory boards for numerous pharmaceutical companies, including Almirall, GSK, AstraZeneca, Novartis and Nycomed. He has received industry-sponsored grants from AstraZeneca, Biomarck, Centocor, Mpex, Nabi, Novartis and Otsuka.

AA has received fees for speaking and consultancy, as well as funds for research, from Almirall, GSK, AstraZeneca, Boehringer Ingelheim, Esteve and Chiesi.

PC has received fees for speaking and consultancy from Centocor, AstraZeneca, Chiesi, GSK, Boehringer Ingelheim, Nycomed, Novartis and Almirall, as well as research grants from Schering Plough and Centocor.

HM has received funds for research and fees for consulting from a number of pharmaceutical companies.

LF has served as a consultant to, and received lecture fees and grant support from, Nycomed, AstraZeneca, Boehringer Ingelheim, Chiesi, GSK, Merck Sharp & Dohme and Novartis.

JFD has served on advisory boards for Almirall and Forest Laboratories.

EDB has received remuneration for consulting and serving on advisory boards for Almirall, and his institution has received grants for taking part in clinical trials sponsored by Almirall and Forest Laboratories.

NJG has received honoraria for presentations on COPD treatment at meetings sponsored by Almirall and Forest Laboratories, as well as payments for advisory board consultations. His institution has received research grants from Almirall and Forest Laboratories.

RL and EGG are employees of Almirall.

CC is an employee of Forest Laboratories, and holds stock and options in the company.

## Authors' contributions

PWJ was the co-ordinating study investigator and SIR, AA, PC, HM, LF, JFD, EDB and NJG were study investigators. All study investigators, CC and EGG made substantial contributions to the conception and design of the study and the interpretation of the study data. RL supervised the statistical analysis. All authors contributed to the development of the manuscript, and read and approved the final version prior to submission.
